# Research on integrated simulation platform for urban traffic control connecting simulation and practice

**DOI:** 10.1038/s41598-022-08481-w

**Published:** 2022-03-16

**Authors:** Lili Zhang, Qi Zhao, Pei Yu, Jing Li, Di Yao, Xinzhe Wang, Li Wang, Lingyu Zhang

**Affiliations:** 1 College of Information Engineering, Beijing Institute of Petrochemical Technology, Beijing, 102617 China; 2 Xinghe Environmental Protection Technology Co., LTD, Zhengzhou, 450001 China; 3 Beijing Key Lab of Urban Intelligent Control Technology, North China University of Technology, Beijing, 100144 China; 4 China Fire and Rescue Academy, Beijing, 102202 China

**Keywords:** Information technology, Software, Civil engineering

## Abstract

Though effective in theoretical simulation, the established traffic control models and optimization algorithms will result in model mismatch or even control strategy failure in actual application. However, they are commonly adopted in traffic signal control research, resulting in the unavailability of many exceptional control algorithms in practice. Simulation should function as a bridge between theoretical research and actual application, allowing the gap between the two to be communicated and made up for. However, an effective connection between the two has yet to be established to enable simulation methods in existing traffic control research. To this end, we designed and developed a simulation platform for "Online Application—HILS (Hardware-in-the-Loop Simulation)—Practice" integration over traffic signal control. In this paper, the architecture and characteristics of the integrated simulation platform were described. Besides, the function of each module of the platform was detailed, followed by listing simulation examples for six complex scenarios, with the active control scenario being selected for simulation comparison analysis. The findings demonstrated extensive road network simulation with the integrated simulation platform, multidimensional control variables, control strategies with support, as well as stable and reliable operation. It can be used to verify several sorts of traffic control simulation with variable dimensions.

## Introduction

As a longstanding academic concern in this field, intelligent urban transportation has witnessed plenty of theoretical and technological innovations and applications^1^, among which traffic control is regarded as the pearl in the crown of intelligent transportation. It has served as a key measure to ease traffic congestion and solve traffic problems. For this reason, advanced traffic signal control systems such as SCOOT^2^, SCATS^3^, and series of excellent traffic control algorithms including model-driven algorithm^4^, data-driven algorithm^5^, and artificial intelligence^6^ have emerged throughout nearly a century of development, which functioned as staunch supporters of the rapid development of urban traffic control.

Using simulation to analyze and verify traffic control algorithms is an important element of traffic control research. Related research in China started in the 1980s, which marked the earliest introduction and use of commercial traffic simulation software for the main, followed by the secondary development based on commercial software such as VISSIM and Paramics to verify control algorithms^7,8^. Control algorithm verification with tools such as Matlab at a given input and output is common in a class of research on traffic control using the control theory^9^. HILS was developed by combining commercial traffic simulation software with traffic control hardware^10^.

Although existing research have yielded fruitful results, the following two issues remained to be addressed:

1. Existing traffic control simulation fails to realize the real-time connection between simulation and actual traffic control on-site, making real-time simulation, verification, and evaluation unavailable.
2. Currently, most traffic control simulation adopts traditional passive traffic control theory as the foundation for the underlying architecture design, which provides insufficient support for advanced control models and algorithms, resulting in the lack of effective and practical simulation results. It presents deficiencies in supporting the simulation of multi-variable and multi-dimensional active traffic control for future ICVs and autonomous driving environments.

Therefore, in traffic control research adopting the above traditional simulation methods, the constructed control models and optimization algorithms often result in problems such as model mismatch and control strategy failure in practice, despite their evident effects in theoretical simulation. Consequently, many excellent traffic control algorithms are impractical as they fail to follow the requirements for the practical application of traffic control.

- The construction of control models, strategies, and optimization methods faces ideal assumptions and broad constrains as various possible problems in the actual scenario are not considered.
- The simulation should serve as a link between theoretical research and practical application, bridging the gap between the two, which has yet to be achieved by the current simulation methods for traffic control.

To address these issues, an "Online Application—HILS—Practice" simulation integration platform for real-time testing and verification of active traffic control has been designed and developed. It established its field traffic controller and detector and online simulation to enable effective connection by directly building a data channel in the field, and the real-time verification for the advanced traffic control algorithm was implemented to ensure security and validity. It enabled the transition from traditional passive traffic control to active traffic control, increased the dimension of control variables via resource definition, and improved the flexibility and control capabilities of traffic control models and strategies.

The following is how the rest of the paper is organized: a literature review of relevant work is presented in “Literature review” and the architecture of the simulation integration platform in “Architecture and characteristics of simulation integration platform”, along with a summary of its characteristics; “Main functions of simulation integration platform” discusses the main functions of the simulation integration platform and how to realize the transition from simulation to reality; “Simulation experiment” presents simulation examples for six complex traffic scenarios to compare and analyze the simulation by active and passive control methods; and the conclusion of this paper and directions for future research are detailed in “Conclusion”.

## Literature review

As an age-old challenge that dates back to the 1950s^11^, intersection control is the most important issue in the field of urban traffic, even in the future vehicle–road collaboration and popular autonomous driving, making it a great concern of many scholars. Traffic simulation functions as an important means of traffic control research as it encourages the rapid development of the field. Two main traffic control simulation methods are currently available:

### Commercial traffic simulation software and other development tools

Microtraffic simulation software is often employed to evaluate the effectiveness of traffic control strategies as it is unsafe and impractical to test them directly in the field^12^. As a result, researchers often collect historical data to perform repeated tests with the simulation software. Once the effectiveness of the control strategy is recognized, the traffic engineer goes through a series of procedures necessary to load it into an operational traffic control system or traffic controller^13^, to avoid possible safety problems. Secondary development modules are available in commonly used commercial traffic simulation software, including VISSIM, CORSIM, TransModeler, and SimTraffic^14–17^. Then an advanced control strategy can be developed to send the detector data generated by the simulation software to the simulator, which outputs the signal state and evaluates the traffic condition based on this information. Some traffic control studies adopted classical control theory, and usually used development tools such as Matlab to verify the rigor of theoretical proof by simple examples^9^.

### Hardware-in-the-Loop (HILS)

The concept of HILS was firstly proposed by Sisle in the 1980s to evaluate the performance of an active missile from prelaunch to interception^18^. The basic idea of this concept was to create a link between traffic controllers and simulation software with a controller interface device (CID), which sent detector data obtained from the simulation software to the physical traffic signal controller. At the same time, the traffic signal controller determined the signal state according to the predetermined signal time control strategy and detector data from the simulation software. The device also transmitted real-time signal state from the physical traffic signal controller to the simulation software^19–23^. Although HILS brought the simulation closer to reality, previous studies have shown that each controller in HILS corresponded to a dedicated CID, and the control strategy in the simulation could not be directly loaded into the field traffic controller through CID.

### Advanced active traffic control

Traditional traffic control has developed over many years^24–26^. However, the abundance of traffic control data benefited from the development of information technology, and the theory of traffic control may therefore be changed. Therefore, some studies have proposed active traffic control. At present, there are two main types of research on active traffic control at intersections: One is the active control based on traffic flow prediction, which is realized by building a traffic flow prediction model combined with traditional traffic control^27–30^. The other is active control based on cooperative vehicle infrastructure, which combines vehicle speed guidance and traffic control to achieve active control through the use of cooperative vehicle infrastructure information^31–35^.

However, to the best of our knowledge, the various traffic simulation methods used in current traffic control research have not effectively linked theoretical research with practical application, with the gap between the two remaining the primary impediment to the practical availability of traffic control algorithms. Considering other practical factors, the original control methods such as time control are still applied in the field.

## Architecture and characteristics of simulation integration platform

### Architecture of simulation integration platform

**Figure 1:**
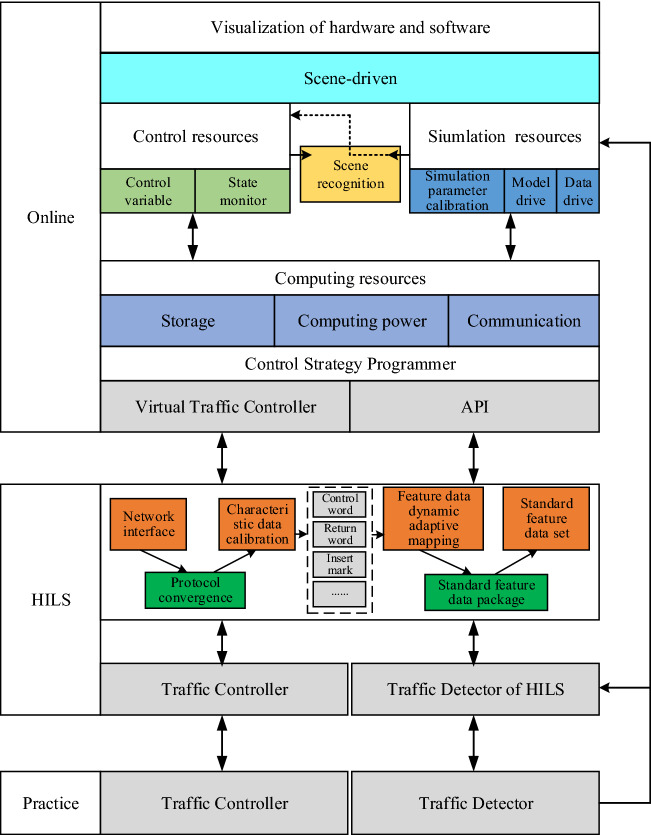
Traffic control simulation integrated platform structure.

The simulation integration platform based on resource description, taking the three levels of simulation, control, and calculation into consideration, defines and encapsulates the three resources to form a resource pool. It can flexibly schedule simulation, control, and computing resources based on demand during simulation verification, as shown in Fig. 1. The simulation integration platform can be divided into three parts. First and foremost, it builds diverse simulation scenarios via a scene-driven approach. The "Online Application" simulation employed control resources to expand and apply control variables. At the same time, on-site detection data was used to calibrate simulation parameters and drive simulation operations. Computing resources enabled resource scheduling to meet storage, computing power, and communication requirements during simulation. Secondly, the underlying virtual controller was connected to the real controller "HILS", and the API effectively encapsulated the control variables and functions. The control strategy programmer was used to rapidly design and develop advanced traffic control methods. Finally, the two traffic controllers, "Practice" and "HILS", were identical. The simulation integration platform allowed simulation to be connected to field applications while ensuring operational safety.

**Figure 2:**
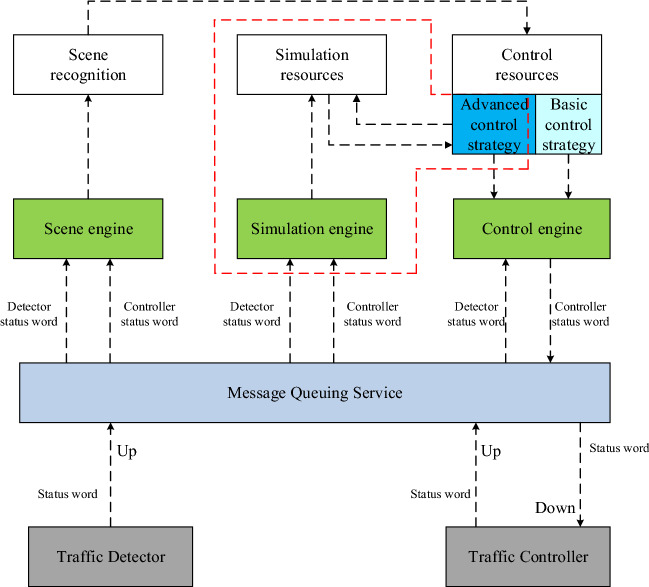
Traffic control simulation integrated platform data stream.

The simulation integration platform was driven by scene, simulation, and control engines. During simulation verification, the scene engine applied to the message queue service to call traffic detector and traffic controller state data. It encapsulated the data according to the input data requirements of the scene recognition algorithm before sending it to the scene recognition module to identify the current traffic scene, and then delivered the identification results to the control strategy module as input. If only the basic control strategy was required for the current traffic scene, the basic control strategy generated control parameters and transmitted them to the control engine, which merged and encapsulated the control parameters and basic parameters before sending them to the traffic controller for execution. If advanced control strategies were required for the current traffic scene, the simulation engine applied to the message queue service to invocate traffic detector and traffic controller state data. It encapsulated the data according to the input data requirements for the simulation before sending it to the simulation engine. At the same time, the advanced control strategy transmitted the generated control parameters to the simulation engine to form a simulation subroutine and obtained a definite control through simulation. The parameters were sent to the control engine, which merged and encapsulated the control parameters and basic parameters before sending them to the traffic controller for execution, as shown in Fig. 2.

### Characteristics of simulation integration platform

The four main characteristics of the simulation integration platform adopting the "Online Application—HILS—Practice" fusion architecture are as follows:

1. **It built a resource-based basic library that allowed for flexible scheduling of three resources, i.e., simulation, control, and calculation.** The nature of changes in road network traffic flow lies in matching traffic demand and the resources of time and space. Traffic control is used to allocate time and space resources on the premise of traffic safety. The resource description-based simulation integration platform took the three levels of simulation, control, and calculation into consideration in a unified manner, defined and encapsulated the three as resources to form a resource pool. It could flexibly schedule simulation, control, and compute resources based on demand during simulation verification. Among them, the control resource mainly referred to the resource definition of control variables in traffic control. Computing resources were applied to meet the requirements including (1) computing power for larger-scale simulation calculations; (2) computing power for advanced control and optimized algorithms; (3) computing power for modeling more complex traffic scenarios; (4) computing power for simultaneous online simulation for multiple users; and (5) requirements for real-time/historical data collection, transmission, and storage.
2. **It enables the dual integration of model-driven and data-driven approaches, with the support of scene-driven approach.** The simulation integration platform can be used to construct complex traffic scenarios and encapsulate basic simulation parameters. It supports both model-driven and data-driven simulations, with the former including car-following models, queuing models, and traffic flow distribution models (using the manual calibration of model parameters and other parameters). The data-driven simulation drives the traffic flow through real-time or historical data, which comes from on-site traffic detection data, and dynamic simulation parameters are calibrated through real data.
3. **It upgraded passive traffic control to active traffic control by increasing the dimension of control variables.** The fundamental problem for urban traffic control lies in the allocation of road time and space resources as it starts from the 'physical resource problem' of ' cars occupying physical space' on the timeline of road traffic. Therefore, the concept of generalized traffic control was taken into account. It transformed the traditional passive traffic control with cycle and green-signal ratio adjustment as the core into a section with controllable vehicle speed, variable lanes, adjustable phase and sequence, and chain-like connection characteristics. Active traffic control was achieved by increasing the dimension of control variables. Due to its diverse control variables, active traffic control could meet current and future traffic control needs in fully automated driving, unmanned driving, vehicle–road coordination, and other complex and special scenarios to a greater extent.
4. **It enabled the high-efficient reachability from control model to control strategy then to controller by bridging the gap between simulation and reality.** (1) Scene setting and parameter calibration were synchronously loaded into the traffic controller for parameter protection in accordance with the requirements of the protection mechanism. (2) The traffic control engine shared the same system with the on-site traffic control system, which could abstract the control variables/language of the traffic controller to form a mapping relationship with those of the simulation control engine, directly driving the traffic through the simulation control engine. By doing so, the designer of the control strategy could be free of focusing on the difference between the simulation controller and the on-site traffic controller. (3) It supported traffic controllers by abstracting and decoupling the relationship between basic protection parameters and control variables, as well as a variety of advanced traffic controllers. (4) The platform leveraged real data to calibrate simulation parameters, allowing the control strategy to be trained and implemented in an environment close to the scene. It could effectively solve the problem that the existing control strategy simulation is effective in theory but cannot be applied in the field. (5) It also freed traffic control engineers by providing control strategy programmer and API, allowing them to focus on the design of the control strategy rather than programming skills.

## Main functions of simulation integration platform

### "Online application" simulation

**Figure 3:**
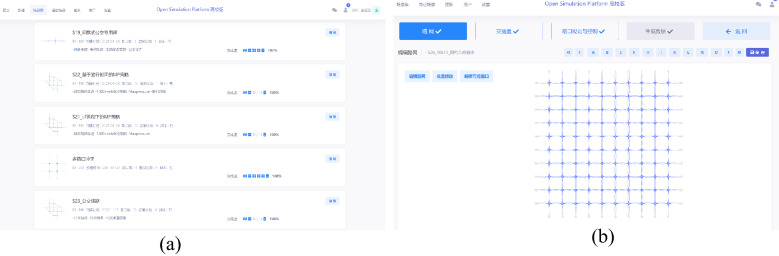
Online simulation module. (**a**) Simulation scene library, (**b**) Simulation parameter setting.

Being designed to quickly analyze and verify new control strategies, the "Online Application" simulation module took the traffic flow from the meso perspective as the main body and the intersections and road sections from the microscopic perspective as the control core, with a focus on the shape of simulated traffic flow under the influence of control strategy, to encourage iterative control strategy optimization. The simulation engine was designed with reference to SUMO^36^ to visualize traffic flow, calibrate dynamic parameters of traffic flow, and basic road network parameters, as illustrated in Fig. 3.

Auto-calibration of simulation parameters: It is important to note that the dynamic characteristics of a single vehicle, as well as the manual calibration of simulation parameters, were intentionally ignored in the design of the simulation system. The system adopted detector data and GIS data to automatically calibrate static data (road properties: road length, lane width, intersection shape, etc.), dynamic data (vehicle type ratio, vehicle type), velocity distribution, steering ratio, headway time, etc. At the same time, traffic flow and car-following characteristics were based on the accumulation of historical detection data, and real-time detection data were corrected.

Architecture of control strategy and API design: The architecture of the simulation integration platform supported the development of advanced control strategies, which consisted of three parts: data preparation, control strategy, and device driver.

**Figure 4:**
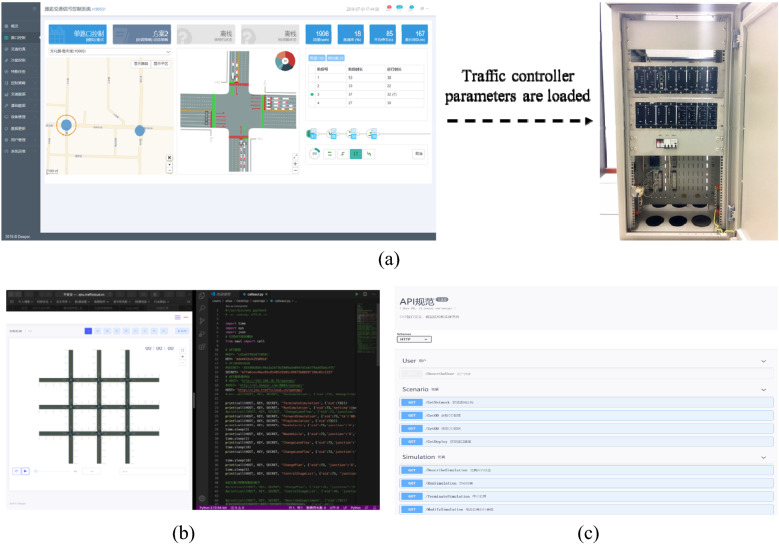
Map of virtual controller to real traffic controller. (**a**) Basic parameter configuration under protection mechanism, (**b**) Control strategy programmer, (**c**) API package.

- In the data preparation part, the test data was encapsulated as standard input and output variables, and the signal control, vehicle, and variable sign were stored as control variables in the database.
- In the control strategy part, various virtual scenes were constructed based on different traffic demands, and control strategies were designed for different scenes. The set of control strategies was called agency, and control strategy was called the agent. That is to say, the new control strategy designed by researchers was defined as an agent, and the corresponding agent run under given conditions in the simulation.
- The device driver connected the simulation and the scene, through which a new control strategy could be directly pushed to the on-site control equipment once it presented to be reliable, allowing the implementation in the real environment at the right time. The virtual controller was used to set the basic parameters of traffic control, which were then loaded into the entity of the traffic controller for basic control. The designed complex traffic control strategy was written by the control strategy programmer, and the traffic controller was driven for execution through API, as shown in Fig. 4.

### "Online application—HILS" parallel simulation

**Figure 5:**
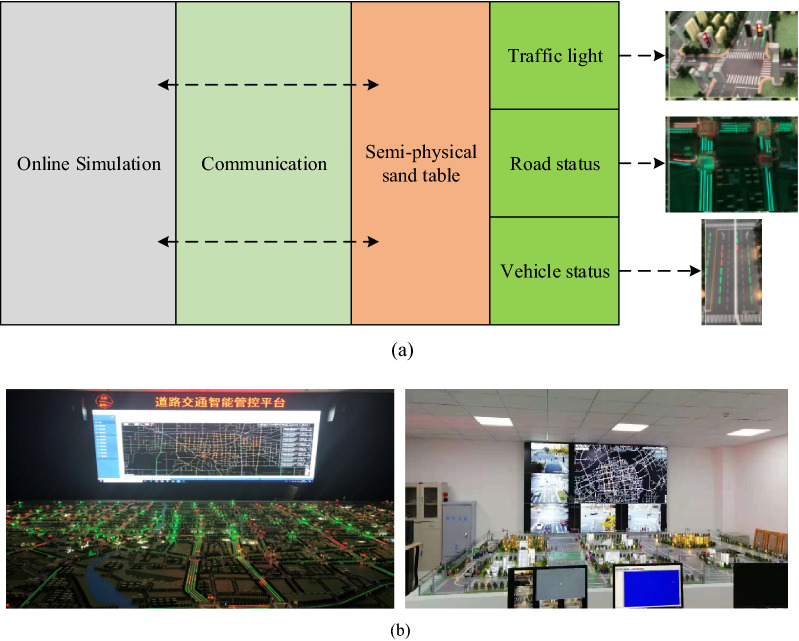
"Online-HILS" parallel simulation. (**a**) "Online-HILS" parallel simulation structure. (**b**) "Online-HILS" parallel simulation display.

As demonstrated in Fig. 5, the "Online Application—HILS" parallel simulation served as a bridge between "Online Application" and "Practice". Being same as the road network in the online simulation, the semi-physical sand table in "HILS" employed a real traffic controller. An "Online Application—HILS" inspection method was formed by accepting the control strategy in the simulation, and the control strategy could be verified indefinitely through the simulation process. The control value obtained in the control strategy was input to the "Practice" traffic controller for execution as the control strategy was verified to be available and safe.

### "Online application—HILS—practice" integration

**Figure 6:**
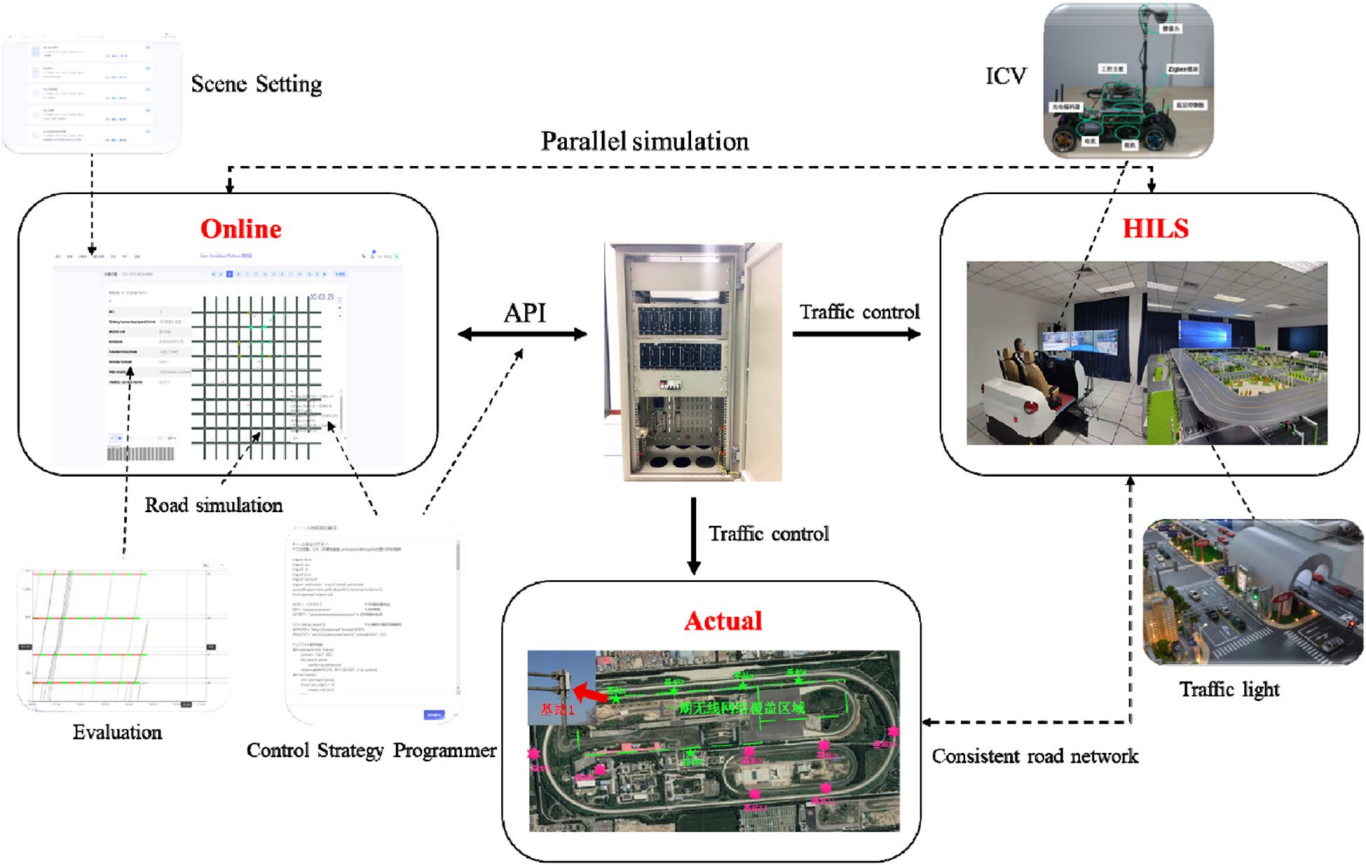
Simulation integrated platform structure.

Figure 6 depicts the " Online Application—HILS—Practice" fusion, which combined "Online Application" simulation, "Online Application—HILS" parallel simulation, as well as actual traffic controllers and detectors. As the most critical technology, the establishment of a unified traffic control engine allowed the seamless connection between simulation and field applications. As the designed control strategy was applied to the actual traffic controller on the field, the actual traffic detection data was transmitted to the simulation for parameter calibration, simulation drive, and control effect evaluation, and finally, a closed-loop verification and correction of the control strategy was formed.

## Simulation experiment

### Examples of complex traffic scenarios and advanced control strategies

**Figure 7:**
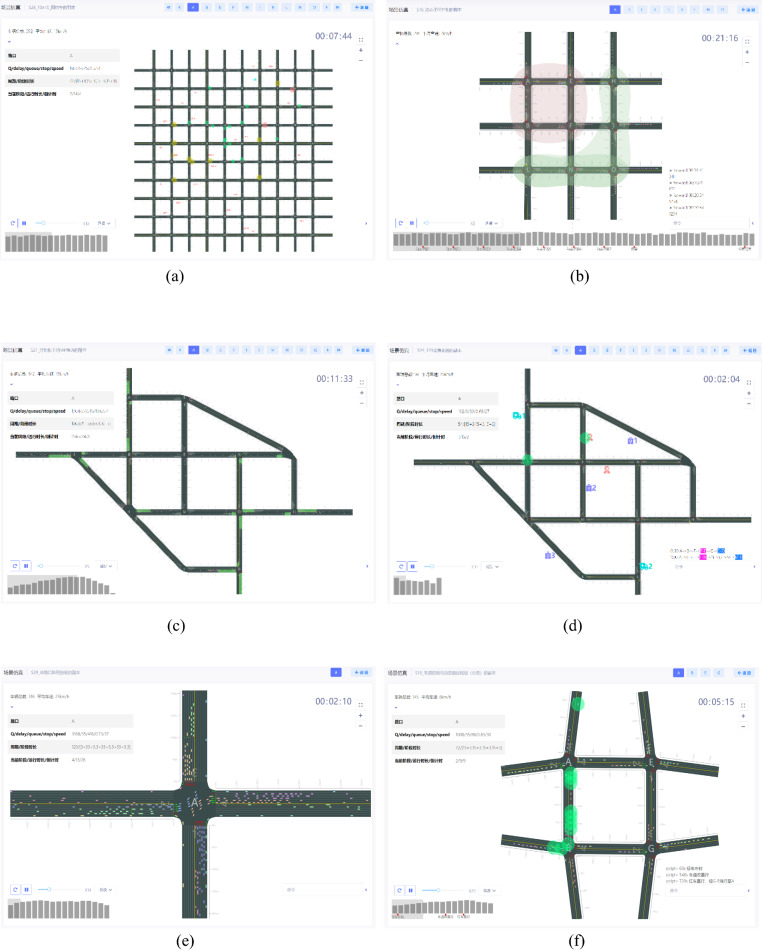
Some examples of the simulation integration platform. (**a**) Example of large-scale road network simulation, (**b**) Example of the dynamic subarea control simulation, (**c**) Example of supersaturation control simulation, (**d**) Example of emergency vehicle priority control simulation, (**e**) Example of queuing and array control, (**f**) Example of the dynamic lane and speed control.

The simulation integration platform covered over 30 complex scenarios and the application of numerous advanced control strategies, including intersection control, area control, vehicle–road coordinated control, special vehicle control, and other trending research directions in the field. The paper presents six complex traffic scenarios in Fig. 7, namely, large-scale road network simulation, dynamic subarea control simulation, over-saturation control simulation, emergency vehicle priority control simulation, line-up control simulation, and active control (dynamic lane and speed control combined control) simulation, involving the application of various control variables such as vehicle speed, lane, phase, and phase sequence.

### Comparative analysis of simulation examples

The comparative analysis of active traffic control^37^ and traditional traffic control^38^ is presented in Fig. 7f, with undersaturated/ oversaturated traffic scene, and the simulation parameters are shown in Table 1.

**Table 1:** Basic parameters of simulation.

Parameter	Content
Road	Road network: 2*2 intersections; the length of the connecting section of the intersections: 470 m–490 m, with six two-way lanes, channelization set at 30 m at the entrance of the intersections; width: 3.5 m
Vehicle	Speed distribution: [20 km/h, 60 km/h], the ratio of vehicle types: 1: 99 (large cars: small cars), the ratio of buses to emergency vehicles: 99:1 in the large car
Flow	Initial: the flow of stage 1: 1000 v/h; the flow of stage 2: 500 v/h; the flow of stage 3: 1000 v/h; the flow of stage 4: 500v/h. Every 3600 s multiplied the flow of each stage by the coefficient of change x, 0.5 < × < 2
Intersection and Signal Control	The intersections were numbered A, B, E, G, the initial plan of the intersection all adopted 4-stage signal control. The stages were stage1: north–south straight; stage2: north–south left; stage3: east–west straight. Stage4: turn left from east to west; straight-right vehicles and straight-going vehicles were released at the same time; the signal control period was 160 s; the interval time was 3 s for yellow light and 2 s for all red; Tstage1: 45 s, Tstage2: 20 s, Tstage3: 45 s, and Tstage4: 20 s
Scene	Undersaturation, oversaturation
Data Collection and	The road section number, lane number, whole road section density, flow, and vehicle speed were collected, with an acquisition interval of 600 s
Evaluation Parameters	The average number of stops and average delay time

**Figure 8:**
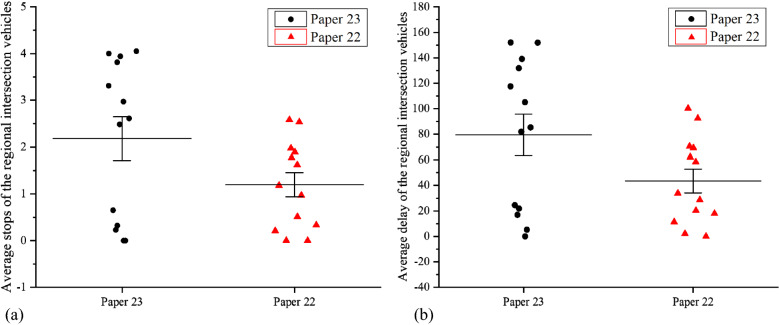
Comparison of control effects. (**a**) Average stops of the regional intersection vehicles, (**b**) Average delay of the regional intersection vehicles.

According to the comparative analysis of simulation under undersaturated and oversaturated scenarios as shown in Fig. 8, undersaturated traffic saw equal control effects of the two control methods; however, as the state of traffic flow changed from undersaturated to oversaturated, the active control method in the paper^34^ had a significantly better control effect than the paper^35^.

## Conclusion

The application of traditional traffic simulation methods in traffic control research often results in the problem that the constructed traffic control models and optimization algorithms face model mismatch and control strategy failure in the actual application, despite their obvious effects in theoretical simulations. This can be explained by: (1) ideal conditions and broad constraints. In the traffic control models, strategies, and optimization methods established in many studies, various possible problems in actual application were not considered; and (2) The existing simulation methods failed to connect theory and practice, with the gap between the two remaining the greatest obstacle to the practicability of the control algorithm, which, along with other basic problems, caused most primitive control methods such as time control and induction control to be unavailable in practice.

Paper^39^ pointed out that ‘In fact, simulation refers to the construction of a system similar to the real world. A controller that is feasible for simulation calculations on this simulation system should have the same effect after being connected to the real control object.’ This paper describes the design and development of an "Online Application—HILS—Practice" simulation integration platform for urban traffic control, which combines practical engineering experience and in-depth consideration of the problems in traffic control simulation. It is based on resource nation and describes simulation, control, and calculation as resources, allowing them to be flexible and schedulable. The dimension of the control variables has been increased, which enabled the output of multiple control variables, including green light, phase, phase sequence, lane, and vehicle speed. On this basis, various functions of "Online" simulation, "Online-HILS" parallel simulation, and "Online-HILS-Practice" integration were studied in detail. The virtual signal machine and standard protocol converter made the traffic controller a bridge connecting the "Online" simulation, "HILS" simulation, and practical applications. Finally, six simulation examples for complex scenarios were listed. The two methods of active control and passive control were simulated, compared, and analyzed with the simulation integration platform under two typical undersaturated and oversaturated scenario. The research results showed that the simulation integration platform can not only perform large-scale road network simulation, but also realize a variety of complex control strategies from vehicle speed control and lane control to phase and phase sequence control, with stable, reliable, and real-time simulation.

## Data Availability

The data provided was for use in this paper only because it involves commercial and government requirements for the use of data.

## References

[1] Zhang LL, Wang L (2019). Architecture of a new generation of artificial intelligence traffic signal controller. J. Chongqing Jiaotong Univ..

[2] Hunt PB, Robertson DI, Bretherton RD (1982). The SCOOT on-line traffic signal optimisation technique. Traffic Eng. Control.

[3] Luk JYK (1984). Two traffic-responsive area traffic control methods: SCAT and SCOOT. Traffic Eng. control.

[4] Yang WC, Zhang L, Tian BJ (2017). Urban adaptive traffic signal control methods: A survey of the state of art. Transp. Sci. Technol..

[5] Hou ZS, Xu JX (2009). On data-driven control theory: The state of the art and perspective. Acta Autom. Sin..

[6] Zhou ZJ (2010). Review on new generation traffic simulation. J. Syst. Simul..

[7] Wang D, Tian ZZ, Yang G (2019). Virtual controller interface device for hardware-in-the-loop simulation of traffic signals. IEEE Intell. Transp. Syst. Mag..

[8] Zhang GW (2014). The application and development of dynamic traffic simulation theory. J. Transp. Syst. Eng. Inform. Technol..

[9] He ZH, Chen YZ, Shi JJ (2013). Stability of switched sever system and signal timing of intersection. Control Theory Appl..

[10] Li P, Michandani PB (2016). A new hardware-in-the-loop traffic signal simulation framework to bridge traffic signal research and practice. IEEE Trans. Intell. Transp. Syst..

[11] Gazis DC (1964). Optimum control of a system of oversaturated intersections. Oper. Res..

[12] Baldi S, Michailidis I, Ntampasi V (2019). A simulation-based traffic signal control for congested urban traffic networks. Transp. Sci..

[13] Xiang C, Salem M, Da ST (2008). Real time software-in-the-loop simulation for control performance validation. SIMULATION.

[14] ITT Systems and Sciences Corporation (1998). CORSIM User’s Manual. Version 1.04.

[15] Koonce P, Urbanik T, Bullock D (1999). Evaluation of diamond interchange signal controller settings by using hardware-in-the-loop simulation. Transp. Res. Record.

[16] Lu L, Yun T, Li L (2010). A comparison of phase transitions produced by PARAMICS, transmodeler, and vissim. IEEE Intell. Transp. Syst. Mag..

[17] ITT Systems and Sciences Corporation (1998). Traffic Software Integrated System Version 4.3a CORSIM Run-Time Extension.

[18] Sisle ME (1982). Hardware-in-the-loop simulation for an active missile. SIMULATION.

[19] Engelbrecht, R., Poe, C., Balke, K. Development of a distributed hardware-in-the-loop simulation system for transportation networks. *Presented at the 78th Annu. Meeting Transportation Research Board*, Washington, D.C., 1999.

[20] Bullock D, Johnson B, Wells RB (2004). Hardware-in-the-loop simulation. Transp. Res. Part C Emerg. Technol..

[21] Bullock D, Catarella A (1998). A real-time simulation environment for evaluating traffic signal systems1. Transp. Res. Record J. Transp. Res. Board.

[22] Chen S, Chen Y, Zhang S (2019). A novel integrated simulation and testing platform for self-driving cars with hardware in the loop. IEEE Trans. Intell. Veh..

[23] Wang, S.Y., Chou, C.L., Chiu, Y.H., *et al.* NCTUns 4.0: An integrated simulation platform for vehicular traffic, communication, and network researches. *2007 IEEE 66th Vehicular Technology Conference *2081–2085, (2007).

[24] Chang TH, Sun GY (2004). Modeling and optimization of an oversaturated signalized network. Transp. Res. Part B.

[25] Dinopoulou V, Christina D, Markos P (2006). Applications of the urban traffic control strategy TUC. Eur. J. Oper. Res..

[26] Liu H, Balke KN, Lin W (2008). A reverse causal-effect modeling approach for signal control of an oversaturated intersection. Transp. Res. Part C.

[27] Placzek B (2014). A self-organizing system for urban traffic control based on predictive interval microscopic model. Eng. Appl. Artif. Intell..

[28] Oda T (2007). Signal control by successive updating of control parameters based on prediction of traffic flow. Electr. Eng. Japan.

[29] Ding H, Zhang WH, Zheng XY (2012). Multi-state flow signal control based on traffic prediction. China J. Highway Transp..

[30] Xu JM, Qi C, Wang DH (2018). Self-adaptive control of isolated intersection based on short-term traffic flow prediction. J. Chongqing Jiaotong Univ..

[31] Abulebdeh G (2002). Integrated adaptive-signal dynamic-speed control of signalized arterials. J. Transp. Eng. ASCE.

[32] Bowen Y, Yizhi W, Jianming H (2013). A traffic efficiency promotion algorithm for urban arterial roads based on speed guidance. Int. Conf. Connect. Veh. Expo.

[33] Chen P, Yang C, Sun J (2018). Dynamic eco-driving speed guidance at signalized intersections: Multivehicle driving simulator based experimental study. J. Adv. Transp..

[34] Nuzzolo A, Comi A (2021). Dynamic optimal travel strategies in intelligent stochastic transit networks. Information.

[35] Yang K, Menendez M, Zheng N (2019). Heterogeneity aware urban traffic control in a connected vehicle environment: A joint framework for congestion pricing and perimeter control. Transp. Res. Part C Emerg. Technol..

[36] Codeca L, Frank R, Faye S (2017). Luxembourg SUMO traffic (LuST) scenario: Traffic demand evaluation. IEEE Intell. Transp. Syst. Mag..

[37] Zhang LL, Wang L, Zhao Q (2021). Research on urban traffic active control in cooperative vehicle infrastructure. J. Adv. Transp..

[38] He ZH, Wang L, Zhang LY, Li D (2016). Positive system model of urban traffic networks and steady-state signal control. Inform. Control.

[39] Huang L, Yang Y, Li ZK (2018). Thoughts on intelligent control. Sci. Sin. Inform..

